# Time-Dependent Changes in Protein Composition of Medial Prefrontal Cortex in Rats with Neuropathic Pain

**DOI:** 10.3390/ijms23020955

**Published:** 2022-01-16

**Authors:** Hana Ujcikova, Dagoberto Robles, Xu Yue, Petr Svoboda, Yeon Sun Lee, Edita Navratilova

**Affiliations:** 1 Department of Pharmacology, University of Arizona, Tucson, AZ 85724, USA; hana.ujcikova@fgu.cas.cz (H.U.); roblesdagoberto@email.arizona.edu (D.R.); xyue@email.arizona.edu (X.Y.); yeon@email.arizona.edu (Y.S.L.); 2 Department of Neurochemistry, Institute of Physiology of the Czech Academy of Sciences, 142 20 Prague, Czech Republic; svobodap@fgu.cas.cz

**Keywords:** neuropathic pain, affective dimension of pain, pain chronification, prefrontal cortex, proteomics

## Abstract

Chronic pain is associated with time-dependent structural and functional reorganization of the prefrontal cortex that may reflect adaptive pain compensatory and/or maladaptive pain-promoting mechanisms. However, the molecular underpinnings of these changes and whether there are time-dependent relationships to pain progression are not well characterized. In this study, we analyzed protein composition in the medial prefrontal cortex (mPFC) of rats at two timepoints after spinal nerve ligation (SNL) using two-dimensional gel electrophoresis (2D-ELFO) and liquid chromatography with tandem mass spectrometry (LC–MS/MS). SNL, but not sham-operated, rats developed persistent tactile allodynia and thermal hyperalgesia, confirming the presence of experimental neuropathic pain. Two weeks after SNL (early timepoint), we identified 11 proteins involved in signal transduction, protein transport, cell homeostasis, metabolism, and apoptosis, as well as heat-shock proteins and chaperones that were upregulated by more than 1.5-fold compared to the sham-operated rats. Interestingly, there were only four significantly altered proteins identified at 8 weeks after SNL (late timepoint). These findings demonstrate extensive time-dependent modifications of protein expression in the rat mPFC under a chronic neuropathic pain state that might underlie the evolution of chronic pain characterized by early pain-compensatory and later aberrant mechanisms.

## 1. Introduction

Chronic pain patients often suffer from negative emotional, affective, and cognitive consequences that cause a substantial burden on their lives. Chronic pain, therefore, represents a multifaceted disease state that differs from acute somatosensory or transient injury-driven pain conditions. Chronic pain has been suggested to result from changes occurring in multiple parts of the neuraxis, including primary afferent nociceptors [1] and in output cells of the spinal dorsal horn through a net loss of inhibition [2,3] or from morphological and functional changes in the cortex and other brain regions [4,5,6,7]. To date, however, the only drugs that have demonstrated clinical efficacy in chronic pain are those with predominant sites of action in the brain, including opioids, reuptake blockers, and gabapentinoids [8,9,10]. For example, in a study of 170 patients with neuropathic pain, 22 days of intrathecal delivery of gabapentin provided no pain relief [11]. Both human and animal studies have consistently implicated the medial prefrontal cortex (mPFC) in pain processing, especially for the emotional and cognitive aspects of pain [7]. Our previous studies in rodents also suggest that morphine and gabapentin might act primarily in the mPFC to alleviate aversive aspects of chronic pain [12,13,14].

The human mPFC typically refers to the dorsolateral prefrontal cortex, the anterior cingulate cortex (ACC), and the orbitofrontal, dorsomedial, and ventromedial cortices, while the rodent mPFC consists of the ACC, and prelimbic and infralimbic cortices. Importantly, the role of mPFC in aversive aspects of pain has been well characterized by both human and rodent studies [9,14,15,16]. The mPFC is also involved in analgesia and pain modulation [16,17]. Activations in this region have been demonstrated in humans during placebo-induced analgesia [18], after relief of neuropathic pain [19,20], and with the termination of a prolonged noxious thermal stimulation [21]. Recent studies in mice with neuropathic pain demonstrated decreased excitability of prelimbic layer V pyramidal neurons [22]. Accordingly, optogenetic activation of prelimbic mPFC inputs to the periaqueductal gray relieved neuropathic pain [23]. These findings suggest that activation of some mPFC neurons during pain may be linked to the engagement of adaptive, pain compensatory mechanisms, as well as placebo analgesia. It was also suggested that chronic pain might result from a failure to engage pain resolution mechanisms rather than from a transition from acute pain [24]. Altogether, neural changes in the mPFC may initially reflect adaptive changes that engage pain compensatory mechanisms that could be lost after pain chronification. Alternatively, or in addition, changes in the mPFC may reflect maladaptive changes that develop over time and contribute to chronic pain.

The ACC within the mPFC has been demonstrated to encode the aversive components of pain and to modulate pain perception through its connections to descending pain pathways [23,25]. Accordingly, microinjection of excitatory amino acids into the rostral ACC of uninjured rats produced conditioned place aversion reminiscent of an aversive pain state [26]. In contrast, rostral ACC lesion abolished pain-induced aversive behavior [15,27,28]. Several ion channels, receptors, kinases, and signal transduction molecules have been found to promote pain-related synaptic plasticity in the mPFC, although a systematic and time-dependent proteomic analysis in the mPFC has not yet been conducted.

Recently, neuroimaging studies of patients with chronic pain have uncovered profound morphological and functional reorganization in these brain regions including changes in the pain-related activity, which could explain the high co-occurrence of emotional and cognitive problems associated with chronic pain [4,5,6,7]. For example, a longitudinal study that followed the same back pain patients over 1 year discovered that brain activity in regions associated with acute pain diminished over time, while the activity in emotion-related brain regions increased [29]. Moreover, a neuroimaging study in rats also demonstrated time-dependent alterations in the ACC and mPFC regions in animals with persistent neuropathic pain [30], confirming that key mechanisms of acute and chronic pain processing in the brain are conserved in rats despite significant cortical differences across species. Collectively, both human and preclinical findings in animals suggest that mPFC brain circuits undergo remodeling that might underlie a hypothesized transition to a chronic maladaptive pain state.

Taken together, we postulated that the transition to a chronic pain state will be reflected by time-dependent changes in protein expression in the mPFC of rats with chronic neuropathic pain. To investigate this possibility, we collected the mPFC encompassing the anterior cingulate, prelimbic, and infralimbic cortices of rats with spinal nerve ligation (SNL)-induced neuropathic pain or sham-operated controls and analyzed protein composition by two-dimensional gel electrophoresis (2D-ELFO) and liquid chromatography with tandem mass spectrometry (LC–MS/MS) at early (2 weeks) and late (8 weeks) timepoints following the surgery. Neuropathic pain state was confirmed over time by measuring tactile allodynia and thermal hyperalgesia.

## 2. Results

### 2.1. Pain Behavior

**Figure 1:**
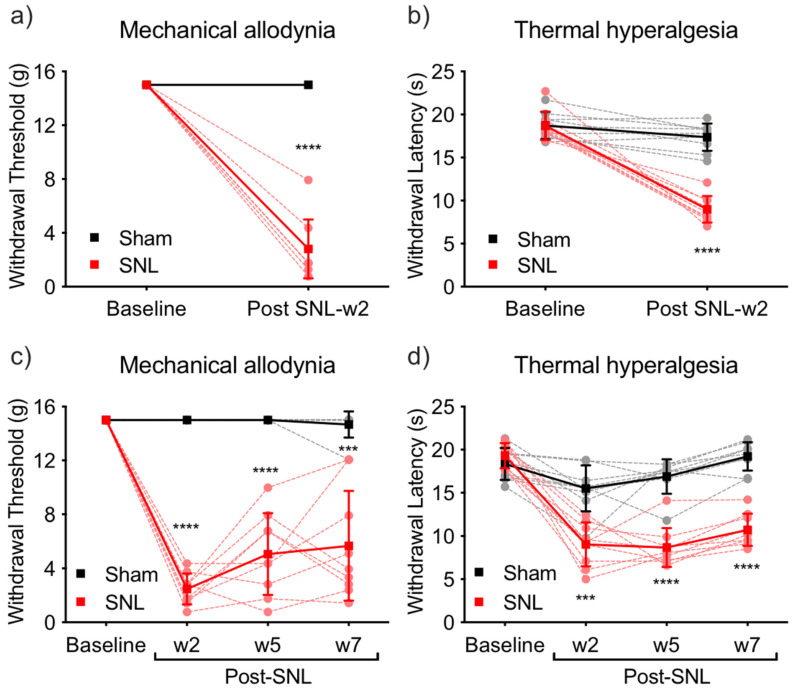
Mechanical and thermal responses for SNL and sham-operated rats: Pain responses of the 2 week groups (nine SNL rats and nine sham rats): (**a**) mechanical allodynia and (**b**) thermal hyperalgesia were measured at baseline (before surgery) and at 2 weeks (w2) post SNL surgery. Pain responses of the 8 week groups (nine SNL rats and nine sham rats): (**c**) mechanical allodynia and (**d**) thermal hyperalgesia were measured at baseline (before surgery) and at 2, 5, and 7 weeks post SNL surgery. Average group data with SEM and individual data are plotted as solid lines and dotted lines, respectively; *** *p* < 0.0005, **** *p* < 0.0001, compared with baseline in the same group.

At 2 weeks following SNL or sham surgery, SNL rats showed significantly lower paw withdrawal thresholds and latencies compared to the baseline, demonstrating the development of tactile allodynia (Figure 1a) and thermal hyperalgesia (Figure 1b), respectively. In contrast, neither mechanical nor thermal withdrawal responses were significantly altered in sham-operated rats at this timepoint. In both tests, two-way RM ANOVA demonstrated a significant effect of time, surgery, and interaction (*p* < 0.0001 for all factors), but not of subject. Post hoc multiple comparisons test showed a significant difference between sham and SNL groups (Figure 1a,b, solid lines; *p* < 0.0001). Every individual SNL rat showed a lower threshold and latency than every individual sham-operated rat (Figure 1a,b, dotted lines).

In another cohort of SNL and sham-operated animals, tactile allodynia and thermal hyperalgesia were evaluated at 2, 5, and 7 week timepoints after the surgery (see methods for details). The SNL group developed long-lasting tactile allodynia and thermal hyperalgesia indicated by significant decreases from baseline in paw withdrawal thresholds and latencies at all timepoints (Figure 1c,d), while the sham-operated rats did not show decreased mechanical or thermal withdrawal responses. Two-way RM ANOVA demonstrated a significant effect of time, surgery, and interaction (*p* < 0.0001 for all factors) on the thermal and tactile responses; there was a significant (*p* = 0.024) effect of subject for the thermal but not tactile responses. Post hoc test demonstrated a significant difference in tactile allodynia and thermal hyperalgesia between sham and SNL groups at all timepoints (Figure 1c,d, solid lines). Every individual SNL rat except one at the 5 week timepoint had lower withdrawal threshold and lower latency than every sham-operated rat, showing the clear group separation (Figure 1c,d, dotted lines).

### 2.2. Identification of Differently Expressed Proteins

#### 2.2.1. Differently Expressed Proteins Identified at 2 Weeks Post SNL

**Figure 2:**
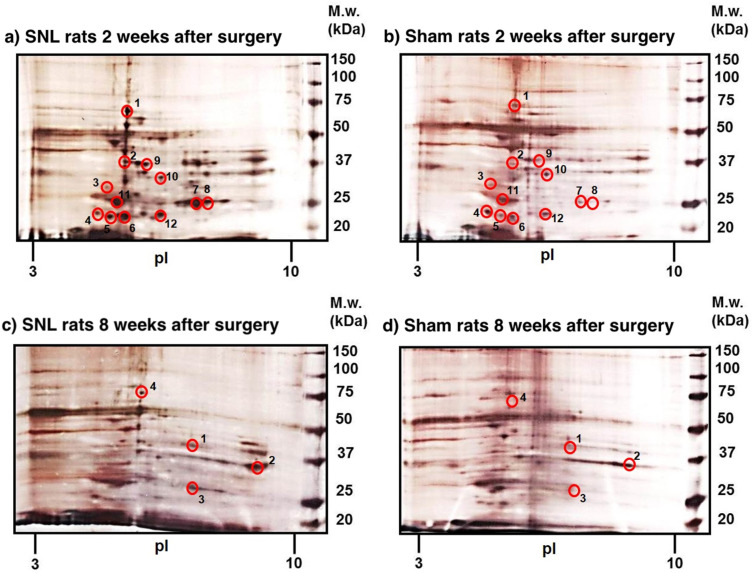
Representative 2D gel maps stained by silver. PNS fractions were isolated from mPFC of rats 2 weeks (**a**,**b**) and 8 weeks (**c**,**d**) after (**a**,**c**) SNL or (**b**,**d**) sham surgery. PNS fractions (250 µg of protein) were resolved by 2D-ELFO and stained by ProteoSilver^TM^ Silver Stain Kit. The differences between SNL and sham-operated rat protein maps were evaluated by PDQuest^TM^ software (Bio-Rad, version 8.0.1). Significantly altered protein spots (*p* < 0.05) are marked with red circles (short-term effect: 1–12; long-term effect: 1–4). The spots cut out of the gels were analyzed by LC–MS/MS (two samples per spot).

**Figure 3:**
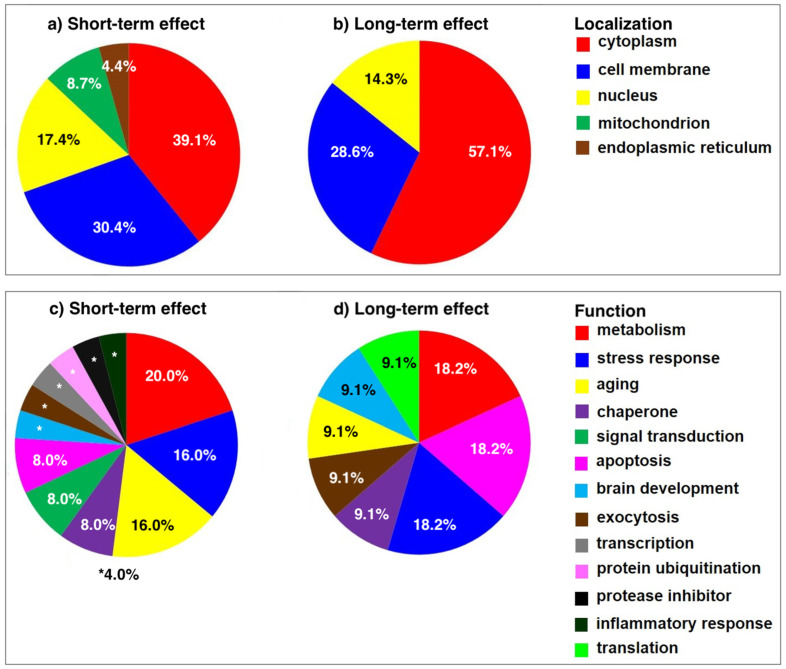
Subcellular localization (**a**,**b**) and functional significance (**c**,**d**) of the proteins upregulated in the mPFC of SNL rats compared to sham-operated rats at 2 weeks (**a**,**c**) and 8 weeks (**b**,**d**) post surgery.

The mPFC tissues from the behaviorally tested SNL and sham rats were collected 2 weeks after the surgery and processed for 2D-ELFO. The silver-stained gels were evaluated by PDQuest software (Bio-Rad) to detect a total of 114 protein spots. Among these, 12 protein spots were recognized as significantly altered (*p* < 0.05) with at least a 1.5-fold difference (Figure 2a,b). LC–MS/MS analysis of the 12 protein spots identified 11 proteins shown in Table 1: heat-shock cognate 71 kDa protein (spot 1), guanine nucleotide-binding protein G(o) subunit alpha (spot 2), 14-3-3 protein epsilon (spot 3), Ras-related protein Rab-3A (spot 4), lactoylglutathione lyase (spot 5), phosphatidylethanolamine-binding protein 1 (spot 6), triosephosphate isomerase (spot 7 and spot 8), isocitrate dehydrogenase (NAD) subunit alpha, mitochondrial precursor (spot 9), malate dehydrogenase, cytoplasmic isoform MDH1 (spot 10), ubiquitin carboxyl-terminal hydrolase isozyme L1 (spot 11), and Parkinson disease protein 7 homolog isoform 2 (spot 12). A complete list of exclusive unique peptides identified by LC–MS/MS analysis is presented in Table S1. The subcellular localization and functional significance of proteins altered in post-nuclear supernatant (PNS) samples of SNL rats were determined according to the current annotations in the UniProt database (https://www.uniprot.org, accessed on 6 January 2022) and the NCBI (https://www.ncbi.nlm.nih.gov, accessed on 6 January 2022) database (Table 2, Figure 3a,b).

#### 2.2.2. Differently Expressed Proteins Identified at 8 Weeks Post SNL

PDQuest analysis of silver-stained gels resolving mPFC samples from SNL and sham-operated rats sacrificed 8 weeks after the surgery identified a total of 106 protein spots. Only four protein spots were recognized as significantly altered (*p* < 0.05) with at least a 1.5-fold difference between two groups (Figure 2c,d) and analyzed by LC–MS/MS (Table 3): peptidyl-prolyl *cis–trans* isomerase D (spot 1), glyceraldehyde-3-phosphate dehydrogenase (spot 2), triosephosphate isomerase (spot 3), and dihydropyrimidinase-related protein 2 isoform X1 (spot 4). A complete list of exclusive unique peptides identified by LC–MS/MS analysis is presented in Table S2. The subcellular localization and functional significance of altered PNS samples of SNL rats were determined according to the current annotations in the UniProt database and the NCBI database (Table 4, Figure 3c,d).

### 2.3. Functional Significance of Altered Proteins

#### 2.3.1. Functional Significance of Proteins Altered at 2 Weeks Post SNL

As shown in Table 2, 14-3-3 protein epsilon was upregulated the most (3.6-fold) 2 weeks after SNL. This phosphoserine and phosphothreonine adapter protein interacts with various targets, including kinases, phosphatases, and transmembrane receptors, and regulates various signaling pathways of receptors, including the mu-opioid receptor [31,32]. Similar to our results, a recent proteomics study [33] reported that 14-3-3 protein epsilon was overexpressed in spinal cord samples collected 10 days after SNL surgery. Although the functional relevance of these changes is unclear, 14-3-3 protein epsilon is likely to promote pain compensatory mechanisms at the early stage of chronic neuropathic pain by regulating endogenous opioid signaling pathways. Alternatively, 14-3-3 protein epsilon may play a role in central sensitization at both spinal and cortical levels to promote the sensory, affective, and cognitive consequences of neuropathic pain.

We also detected a significantly increased level of guanine nucleotide-binding protein G(o) alpha subunit (Gα_o_, 2.4-fold, Table 1). The Gα_o_ protein is constitutively expressed at high levels in mammalian brain and mediates the signal transfer from G-protein-coupled receptors (GPCRs), including opioid receptors through intracellular signal transduction pathways [34]. Importantly, Gα_o_ is 5–10-fold more abundant than other Gα proteins in the brain where it constitutes 5–10% of total membrane proteins [35]. Thus, the 2.4-fold upregulation of Gα_o_ indicates a substantial increase in Gα_o_ signaling in rats with neuropathic pain. The increased level of Gα_o_ was also reported in spinal cord dorsal horn after L5 spinal nerve ligation [36]. It is likely that upregulation of Gα_o_ at early timepoints after injury stimulates the signaling of the mu-opioid receptors, possibly through the endogenous opioid analgesic mechanisms. However, upregulation of Gα_o_ in spinal and cortical regions may also promote pain sensitization through other GPCRs.

The magnitude of upregulation of Ras-related protein Rab-3A (1.5-fold, Table 2) is comparable to upregulation (1.5- and 2.1-fold) observed in the brainstem tissues of SNL rats [37] and in the right central nucleus of the amygdala following the spinal nerve transection [38], respectively. In general, GTPases of the Rab family regulate membrane trafficking, including formation of vesicles, vesicle trafficking, and membrane fusion. Recently, Rab-3A was demonstrated to be critical for exocytosis of dense core vesicles and release of neuromodulators suggesting a role in neuronal transmission [39].

Heat-shock proteins (Hsps) participate in stabilization of a proper protein conformation in various tissues and protect cells from moderate stress. In our mPFC samples, we detected upregulation of heat-shock cognate 71 kDa protein (Hsc71, 2.7-fold) at 2 weeks post SNL. An increase in Hsc71 level was also observed in spinal cord samples from L5 SNL [36], L5-L6 SNL [33], and chronic constriction injury animals [40]. The signaling mediated by the Hsp may represent a basic mechanism of defense against proteotoxic impairment of cell homeostasis [41]. Numerous proteins involved in cellular metabolism and homeostasis have been reported in nerve injury models [40,42]. Our proteomic analysis also revealed the increases in glycolytic enzyme triosephosphate isomerase in two spots (2.3- and 3.1-fold for spot 7 and spot 8, respectively) and enzymes of the Krebs cycle: isocitrate dehydrogenase (NAD) subunit alpha (1.6-fold) and malate dehydrogenase (2.5-fold). The upregulation of metabolic enzymes after the nerve injury may be important for building up cellular defense mechanisms or for activation of metabolic pathways that are normally inactive under steady-state conditions [43]. In addition to the enzymes, the upregulation of phosphatidylethanolamine-binding protein 1 (PEBP-1) was detected. This protease inhibitor participates in the oxidative stress response.

Apoptosis was found to be functionally related to the induction of neuronal sensitization and loss of central inhibitory systems, and both processes were suggested to be linked to the development of neuropathic pain [44]. Our LC–MS/MS analysis identified the increase in lactoylglutathione lyase (1.9-fold) and Parkinson disease protein 7 homolog isoform 2 (3.2-fold), which are involved in negative regulation of apoptosis [45,46]. Ubiquitin carboxyl-terminal hydrolase isozyme L1, which plays a role in protecting cells under stress conditions, was also found to be upregulated (2.8-fold). A study suggested that degradation of selective protein networks by the ubiquitin proteasome system in the spinal cord may lead to the onset of neuropathic and inflammatory pain [33].

#### 2.3.2. Functional Significance of Proteins Altered at 8 Weeks Post SNL

LC–MS/MS analysis identified that fewer proteins (four proteins) were upregulated at 8 weeks compared to 2 weeks after SNL (Table 3). Peptidyl-prolyl *cis–trans* isomerase D upregulated by 2.1-fold is known to be involved in protein folding, negative regulation of apoptosis, and response to oxidative stress [47]. Glyceraldehyde-3-phosphate dehydrogenase upregulated by 2.1-fold is a glycolytic enzyme, and its post-translational modifications may be functionally linked to oxidative stress and apoptosis [48]. Upregulation of another glycolytic enzyme, triosephosphate isomerase, was detected in the mPFC at both 2 weeks (spots 7 (2.3-fold) and 8 (3.1-fold) in Figure 2a,b) and 8 weeks after SNL (spot 3 (2.0-fold) in Figure 2c,d). The persistence of triosephosphate isomerase upregulation from 2 to 8 weeks after development of neuropathic pain (compare Table 1, Table 2, Table 3 and Table 4) indicates that this enzyme is involved in both short- and long-term neuropathic pain states. Dihydropyrimidinase-related protein 2 isoform X1, a developmental protein, upregulated by 2.2-fold has been shown to play a role in remodeling of cytoskeleton after nerve injury [49].

## 3. Discussion

In this study, we focused on pain-induced changes in protein composition in the mPFC that has been demonstrated to play a role in a wide range of functions, including the processing of sensory and affective aspects of pain, cognitive evaluation, and modulation of pain in both human and animal studies. In rodents, immunostaining studies of activity-dependent early genes provided strong evidence of neuronal activation in the mPFC in response to acute and persistent pain [50]. Electrophysiological studies showing long-term potentiation in mPFC slices of injured animals further supported the conclusion that mPFC hyperexcitability contributes to chronic pain [51]. Although several molecules for ion channels, receptors, kinases, and signal transduction have been found to promote pain-related synaptic plasticity in the mPFC, a systematic and time-dependent proteomic analysis in the mPFC has not yet been conducted. Therefore, to investigate the time-related progression of molecular mechanisms in the mPFC during chronic pain, we conducted proteomic analyses of mPFC samples at early (2 weeks) and late (8 weeks) timepoints after induction of chronic neuropathic pain.

We performed 2D-ELFO, the most commonly used method in proteomics, to analyze protein changes in the mPFC of rats at early and late timepoints following SNL surgery. Despite known problems associated with poor detection of proteins that are expressed in very small amounts (such as GPCR) or those exhibiting a low molecular mass, a low isoelectric point, or a high degree of hydrophobicity [52,53,54,55], gel-based proteomics represents a useful tool for identification of pain-related changes in protein expression [44]. We applied the high-sensitivity and low-background silver stain ProteoSilver^TM^ method that is compatible with mass spectrometry analysis. To the best of our knowledge, this is the first proteomic comparison of the mPFC of SNL-treated and sham-operated rats at early and late timepoints following peripheral nerve injury-induced pain.

The Hargreaves and von Frey tests clearly showed that SNL rats established persistent neuropathic pain: thermal hyperalgesia and tactile allodynia (Figure 1). Our previous studies showed that approximately 10% [56] of male Sprague-Dawley rats are resilient to developing SNL-induced peripheral hypersensitivity due to efficient descending pain inhibition from the brain. We also showed that, in resilient SNL rats, these mechanisms ultimately engage the rostral ventromedial medulla (RVM) [56], while other studies additionally identified a role for the ACC and mPFC projections to periaqueductal gray (PAG) and RVM in pain modulation [23]. This descending pain-protective mechanism has been characterized in a majority of healthy volunteers [57,58], partially explaining why most people do not develop chronic neuropathic pain after nerve injury. However, the descending pain inhibition is inefficient in some individuals that are prospectively more likely to develop chronic pain after a scheduled surgical procedure [59]. To avoid confounding factors from possible neural adaptations in the mPFC that may exist in resilient animals, and to analyze the same number of rats in each group (*n* = 9), we chose the highest pain responders from the SNL groups (18 out of 25 rats). Likewise, we excluded four out of 22 rats that showed partial hypersensitivity following sham surgery.

Proteomic analysis of silver-stained spots in 2D gels identified two different sets of proteins that were altered during the short- and long-term periods, respectively. At 2 weeks after surgery, 12 protein spots were found significantly altered in SNL rats compared to sham-operated rats. Notable among these proteins was the upregulation of signal transduction molecules: 14-3-3 protein epsilon, Gα_o_ subunit of an inhibitory G protein, and a small GTPase, Ras-related protein Rab-3A. These findings suggest increased neural signaling in the mPFC, which may be consistent with findings that glutamatergic activation of ACC promotes aversive behavior [26], and that lesions of the ACC relieve ongoing neuropathic pain [28]. The increased neural signaling in the mPFC may also reflect the engagement of endogenous pain compensatory mechanisms at the early timepoints during the injury. Other proteins identified by the analysis were those involved in cellular metabolism, stress, apoptosis, and the ubiquitin proteasome pathway. These cellular functions may serve protective roles against increased cellular stress during the early (subacute) neuropathic pain.

At 8 weeks, however, only four protein spots were found to differ significantly between SNL and sham-operated rats. These proteins included chaperones and proteins involved in apoptosis and remodeling of the cytoskeleton. Interestingly, one of these proteins, dihydropyrimidinase-related protein 2 (also known as collapsin response mediator protein-2, or CRMP-2) is known for its function in several pathophysiological and disease states including chronic pain [60]. CRMP-2 is highly expressed in the nervous system and was shown to interact with the presynaptic N-type voltage-gated calcium channel (Cav2.2) in sensory neurons facilitating nociceptive transmission at the spinal level and promoting neuropathic pain [61]. A study found that, in rats, repeated cocaine self-administration resulted in dynamic alterations in the PFC expression of CRMP2 and CaV2.2, suggesting that neuroadaptations in the CRMP2/CaV2.2 signaling in the PFC facilitate drug-seeking behavior [62]. It is plausible that similar CRMP2 sensitization mechanisms may take place in the mPFC during a long-term period to maintain chronic pain.

A comparison of proteins altered at the early and late timepoints indicated that the mPFC undergoes significant and time-dependent remodeling during the progression of ongoing neuropathic pain. This interpretation is consistent with the dynamic reorganization of the mPFC function during pain chronification that has been observed in longitudinal neuroimaging studies in both rats and humans [5,30,63,64]. Only one protein, triosephosphate isomerase, was observed to be persistently upregulated at both timepoints. It may be speculated that at least some protein adaptations in the early time period are related to pain compensatory mechanisms involving endogenous opioid signaling. These early adaptive mechanisms appear to be lost during pain chronification. At the same time, maladaptive changes develop that facilitate the maintenance of chronic pain and the emergence of affective and cognitive comorbidities.

In conclusion, our study demonstrated that protein expressions in rat mPFC were time-dependently altered in response to persistent and chronic neuropathic pain. The alterations involved proteins for signal transduction, cellular metabolism, and remodeling of the cytoskeleton, suggesting that these adaptations may underlie synaptic plasticity and functional changes to modulate pain behavior. Our findings that the changes in mPFC protein composition occurred over time after nerve injury are consistent with neural reorganization observed during pain chronification in patients [6] and may reflect pain-compensatory or pain-promoting mechanisms.

## 4. Materials and Methods

### 4.1. Chemicals

The 10% Mini-Protean^®^Protein Gels 7 cm IPG/prep well, IPG ReadyStrip^TM^ pH 3–10, Bio-Lyte^®^ 3–10 Buffer, Mineral Oil, and Precision Plus Protein^TM^ were purchased from Bio-Rad (Hercules, CA, USA). The Complete^TM^ Protease Inhibitor Cocktail and Pierce^TM^ BCA Protein Assay Kit were purchased from Roche (Indianapolis, IN, USA) and Thermo Fisher Scientific (Waltham, MA, USA), respectively. The Trypsin/Lysine-C mix and ProteaseMax Surfactant trypsin enhancer were purchased from Promega Corporation (Madison, WI, USA). All other chemicals, including ProteoSilver^TM^ Silver Stain Kit, were purchased from Sigma-Aldrich (St. Louis, MO, USA).

### 4.2. Animals

Male Sprague-Dawley rats, 250–300 g, were obtained from Harlan Laboratories (Indianapolis, IN, USA). Animals were group-housed on a 12 h/12 h light/dark cycle. Food and water were available ad libitum. All described procedures received approval from the Institutional Animal Care and Use Committee (IACUC) of the University of Arizona (15-589). Animals were monitored throughout the study following the International Association for the Study of Pain ethical guidelines. Investigators for all behavioral experiments were blinded to the treatment groups.

### 4.3. Surgeries

SNL surgery was performed as described previously [65]. Rats were maintained under 2% *v/v* isoflurane anesthesia delivered in a 3:2 ratio of nitrous oxide and oxygen. A paraspinal incision was made, and the left tail muscle was excised. Part of the L5 transverse process was removed to expose the L5 and L6 spinal nerves, which were then isolated and ligated with a nonabsorbable 6-0 braided silk thread proximal to the formation of the sciatic nerve. The surrounding skin and muscles were closed with absorbable 3-0 sutures. Sham surgery was also performed in an identical manner skipping the ligation step. All rats were monitored for normal behaviors (grooming and mobility), general health, and weight gain after the surgery.

### 4.4. Behavioral Tests

#### 4.4.1. Von Frey Tests

Paw-withdrawal thresholds were measured by von Frey tests. Rats were placed in suspended chambers with wire mesh floors for 30 min to habituate before the test. A series of calibrated von Frey filaments (Stoelting, Wood Dale, IL, USA) in logarithmically spaced increments ranging from 0.41 to 15 g (4–150 N) were applied perpendicularly to the plantar surface of the ipsilateral hind paw until the filament buckled. Withdrawal threshold was determined by sequentially increasing and decreasing the stimulus strength (“up and down” method), analyzed using a Dixon nonparametric test, and expressed as the mean withdrawal threshold [66].

#### 4.4.2. Hargreaves Tests

Paw-withdrawal latencies were measured by Hargreaves tests [67]. Rats were allowed to acclimate in Plexiglas enclosures with raised glass floors for 30 min before the test. After acclimation, a radiant heat source (115 V, UGO Basile Biological Research Apparatus Model 7371, Comerio VA, Italy) was positioned under the glass floor directly beneath the hind paw. The radiant heat source was activated with a reaction time counter (timer), and paw-withdrawal latency was determined by a motion-sensitive detector source that halted both the timer and the heat source when the hind paw was withdrawn. The withdrawal latency to the nearest 0.1 s was automatically recorded. Baseline latencies were established at 17 to 25 s to allow a sufficient window for the detection of possible hyperalgesia. A maximal cutoff of 33 s was used to prevent tissue damage.

#### 4.4.3. Test Groups

A total of 48 rats were randomly assigned to four groups: (1) sham/2 week (11 rats); (2) SNL/2 week (13 rats); (3) sham/8 week (11 rats); 4) SNL/8 week (13 rats). Baseline tactile and thermal responses for groups 1 and 2 and groups 3 and 4 were measured on two consecutive days, and surgeries were performed 1 day after baseline testing. A total of three rats were removed before the tests because of low baseline thresholds or death during the surgery. In the 2 week groups, mechanical and thermal hypersensitivities were evaluated on day 12 or 13. For the protein analysis, two groups of mPFC tissue were collected from nine sham rats with the highest post-surgical withdrawal latencies and nine SNL rats with the lowest post-surgical withdrawal latencies, respectively. The tissues were harvested on day 18 (2 week groups) after the surgery. Rats in the 8 week groups were evaluated for mechanical and thermal hypersensitivity on days 12/13 (2 weeks) and again on days 38 (5 weeks) and 52 (7 weeks) after the surgery. For the protein analysis, two groups of mPFC tissue were collected from nine sham rats with the highest post-surgical withdrawal latencies and nine SNL rats with the lowest post-surgical withdrawal latencies, respectively. The tissues were harvested on day 59 (8 week groups).

#### 4.4.4. Statistical Analysis

All graphs were created and statistical analyses were performed in GraphPad Prism 9. Two-way repeated-measures ANOVA was performed with time after the surgery as the within-subject factor and the surgery as the between-subject factor. Sidak’s multiple-comparisons test was performed to assess the differences between the groups. Significance was set at *p* < 0.05.

### 4.5. Preparation of PNS Fraction from mPFC Tissues of SNL/Sham-Operated Rats

For the collection of mPFC tissue, a 3 mm coronal slice of the brain tissue approximately between Bregma +3.5 mm and Bregma +0.5 mm was cut, and a 2 mm midline section (1 mm on each side) above the corpus callosum was extracted. The mPFC tissues from three animals within the same group were pooled into one sample of approximately 0.1 g: three sham/2 week, three sham/8 week, three SNL/2 week, and three SNL/8 week samples. The tissue was diluted with STEM medium (250 mM sucrose, 20 mM Tris-HCl, 1 mM ethylenediamine tetraacetic acid (EDTA), 3 mM MgCl_2_, pH 7.6) containing 1 mM fresh phenylmethylsulfonyl fluoride and protease inhibitor cocktail, homogenized mildly in a loosely fitting Teflon–glass homogenizer for 7 min (0.1 g wet weight per 0.5 mL), and centrifuged for 5 min at 3500 rpm. PNS samples were immediately analyzed for protein concentration using Pierce^TM^ BCA Protein Assay Kit (Thermo Scientific, Waltham, MA, USA) and were then stored at −80 °C. More PNS fractions were prepared in the same manner approximately 8 weeks after the surgery.

### 4.6. 2D-ELFO

#### 4.6.1. Sample Preparation for Isoelectric Focusing (IEF)

Samples of PNS containing 250 µg of protein were precipitated with ice-cold acetone overnight at −20 °C. After centrifugation at 12,000 rpm for 20 min at 4 °C, the supernatant was removed, and the pellet was precipitated with ice-cold 6% trichloroacetic acid for 1.5 h in an ice bath. After the second centrifugation at 12,000 rpm for 10 min at 4 °C, the supernatant was discarded, and the pellet was washed with 200 μL of ice-cold 96% ethanol for 1 h at room temperature. The mixture was centrifuged at 12,000 rpm for 10 min at 4 °C, and the remaining pellet was dissolved in 140 μL of IEF sample buffer containing 7 M urea, 2 M thiourea, 4% 3-((3-cholamidopropyl)dimethylammonio)-1-propanesulfonic acid, 1% dithiothreitol (DTT), 0.2% ampholines pH 3–10, and 0.01% bromophenol blue for 3 h at room temperature. After a brief centrifugation (12,000 rpm, 1 min), samples were transferred into a groove of the rehydration tray. Immobilized pH gradient (IPG) strips pH 3–10 were placed into the rehydration tray, and the samples were rehydrated overnight. IEF was performed using the Protean^®^ IEF cell system (Bio-Rad, Hercules, CA, USA) under mineral oil at 15 °C in the following manner: 250 V for 20 min, linear gradient; 500 V for 1 h, linear gradient; 1000 V for 1 h, linear gradient; 4000 V for 1 h, linear gradient; 4000 V for 20,000 Vh, rapid gradient. The focused strips were stored at −20 °C or immediately used.

#### 4.6.2. Equilibration of IPG Strips and SDS-PAGE

The focused strips were rinsed thoroughly with ultrapure water, equilibrated in 4 mL of equilibration buffer (50 mM Tris-HCl pH 6.8, 6 M urea, 0.1 mM EDTA, 2% sodium dodecyl sulfate, 30% glycerol, and 0.01% bromophenol blue) containing 1% DTT for 20 min to reduce disulfide bridges and other oxidized groups. Subsequently, the strips were alkylated in an equilibration buffer containing 2.5% iodoacetamide for 20 min. Precision Plus Protein Standard markers were loaded onto a piece of filter paper and placed close to the alkaline side of the strip. The strip and paper containing protein markers were covered with 0.5% agarose. Gels were run in the Mini-Protean Tetra Cell system at 50 V for 5 min and 150 V for approximately 45 min until the bromophenol blue dye reached the end of the gel. At least nine 2D gels were performed with PNS samples prepared from each test group.

### 4.7. Silver Staining

Silver staining was performed using ProteoSilver^TM^ Silver Stain Kit (Sigma-Aldrich, St. Louis, MO, USA) according to the manufacturer’s instructions [68,69]. Briefly, a gel was fixed in 50% ethanol/10% acetic acid for 2 h and then washed with 30% ethanol for 10 min followed by a 10 min wash with 200 mL of ultrapure water. The gel was incubated for 10 min with 1% sensitizer solution and washed twice with 200 mL of ultrapure water for 10 min. The gel was submerged in 1% silver solution for 10 min, washed with 200 mL of ultrapure water for 1 min, and developed with 100 mL of the developer solution until desired intensity of spots was attained. The ProteoSilver Stop solution was added to the developer solution, and gel was incubated for 5 min. All steps were carried out at room temperature on an orbital shaker at 60 to 70 rpm. The gel was stored in 1% acetic acid at 4 °C.

### 4.8. Image Analysis

The stained 2D gels were scanned and quantified by PDQuest 2D Analysis Software (Bio-Rad, version 8.0.1). The process included spot detection, gel matching, and spot quantification. All matched and unmatched spots were then checked manually. First, 2D gels prepared from the mPFC samples of SNL or sham rats 2 weeks after the surgery were evaluated. Second, 2D gels of the mPFC samples collected 8 weeks after SNL or sham surgery were analyzed. Protein spots showing significant quantitative differences of at least 1.5-fold (*p* < 0.05, Student’s *t*-test) were manually cut out from the gels for the analyses.

### 4.9. Identification of Proteins

Protein identification was performed by the Proteomics Core of the University of Arizona (https://proteomics.arizona.edu, accessed on 20 December 2021). The silver-stained gel spots were destained with 30 mM sodium ferricyanide and 100 mM sodium thiosulfate. Disulfide bonds were reduced with DTT, and the resulting free cysteines were alkylated with iodoacetamide before overnight digestion in 50 mM ammonium bicarbonate (pH 7.8), containing 20 ng/μL trypsin/lysine C and ProteaseMax Surfactant trypsin enhancer, at 37 °C. LC–MS/MS analyses were carried out using a Q Exactive Plus mass spectrometer (Thermo Fisher Scientific, San Jose, CA, USA) equipped with a nanoESI source. Peptides were eluted from an Acclaim PepMap 100 trap column (3 μm, 75 μm ID × 25 mm, Thermo Scientific) onto an Acclaim PepMap RSLC analytical column (2 μm, 75 μm ID × 15 cm, Thermo Scientific) using a 5–20% gradient of solvent B (acetonitrile, 0.1% formic acid) over 50 min, 20–50% solvent B over 5 min, 50–95% of solvent B over 3 min, 95% hold of solvent B for 5 min, and finally a return to 5% solvent B in 1 min and another 10 min hold of 5% solvent B. Solvent A consisted of 0.1% formic acid in water. All flow rates were 300 nL/min using a Dionex Ultimate 3000 RSLCnano System (Thermo Scientific). Data-dependent scanning was performed by the Xcalibur v 4.0.27.19 software [70] using a survey scan at 70,000 resolution scanning mass/charge (*m/z*) 353–1550 at an automatic gain control (AGC) target of 1 × 10^5^ and a maximum injection time (IT) of 65 ms, followed by higher-energy collisional dissociation (HCD) tandem mass spectrometry (MS/MS) at 27 normalized collision energy (NCE), of the 10 most intense ions at a resolution of 17,500, an isolation width of 1.5 *m*/*z*, an AGC of 1 × 10^5^, and a maximum IT of 65 ms [70,71,72]. Dynamic exclusion was set to place any selected *m*/*z* on an exclusion list for 20 s after a single MS/MS. Ions of charge state +1, 7, 8, >8, and unassigned were excluded from MS/MS, as were isotopes. Tandem mass spectra were searched against the *Rattus norvegicus* protein database from NCBI (13 November 2018, 83,019 sequences) to which additional common contaminant proteins (e.g., trypsin, keratins; obtained at fttps://ftp.thegpm.org/fasta/cRAP, accessed on 13 November 2018) were appended. All MS/MS spectra were searched using Thermo Proteome Discoverer v 2.2.0388 (Thermo Fisher Scientific) considering fully tryptic peptides with up to two missed cleavage sites. Variable modifications considered during the search included methionine oxidation (15.995 Da), and cysteine carbamidomethylation (57.021 Da). Proteins were identified at 99% confidence with XCorr score cutoffs [72] as determined by a reversed database search. The protein and peptide identification results were also visualized with Scaffold Q + S v 4.8.7 (Proteome Software Inc., Portland OR), a program that relies on various search engine results (e.g., Sequest, X!Tandem, MASCOT) and which uses Bayesian statistics to reliably identify more spectra [71]. Protein identifications were accepted that passed a minimum of two peptides identified at a 0.1% peptide false discovery rate and 90–99.9% protein confidence by the Protein Profit algorithm within Scaffold.

## Figures and Tables

**Table 1:** LC–MS/MS analysis of altered protein spots in PNS fractions isolated from mPFC of SNL versus sham-operated rats 2 weeks after the surgery. The complete list of exclusive unique peptides is presented in Table S1.

Spot	Accession Number	Protein	Exclusive Unique Peptides	SC ^a^ (%)	MW (kDa)	pI ^b^	Change ^c^ (Fold)	*p*-Value
1	NP_077327.1	Heat-shock cognate 71 kDa protein	23	69	71	5.47	↑2.7	0.0398
2	NP_059023.1	Guanine nucleotide-binding protein G(o) subunit alpha	16	58	40	5.48	↑2.4	0.0054
3	P62260.1	14-3-3 protein epsilon	18	84	29	4.86	↑3.6	0.0204
4	NP_037150.2	Ras-related protein Rab-3A	6	49	25	4.73	↑1.5	0.0277
5	NP_997477.1	Lactoylglutathione lyase	10	56	21	5.13	↑1.9	0.0191
6	NP_058932.1	Phosphatidylethanolamine-binding protein 1	8	62	21	5.47	↑1.8	0.0340
7	NP_075211.2	Triosephosphate isomerase	14	71	27	7.37	↑2.3	0.0297
8	NP_075211.2	Triosephosphate isomerase	13	64	27	7.59	↑3.1	0.0044
9	NP_446090.1	Isocitrate dehydrogenase (NAD) subunit alpha, mitochondrial precursor	15	46	40	6.13	↑1.6	0.0116
10	NP_150238.1	Malate dehydrogenase, cytoplasmic isoform MDH1	10	50	37	6.41	↑2.5	0.0326
11	NP_058933.2	Ubiquitin carboxyl-terminal hydrolase isozyme L1	15	87	25	5.24	↑2.8	0.0110
12	NP_476484.1	Parkinson disease protein 7 homolog isoform 2	9	72	20	6.73	↑3.2	0.0352

^a^ Sequence coverage; ^b^ isoelectric point; ^c^ in SNL rats compared to sham rats.

**Table 2:** Subcellular localization and function of altered proteins identified in mPFC isolated from SNL versus sham-operated rats 2 weeks after the surgery.

Accession Number	Protein	Subcellular Localization	Molecular Functions and Biological Processes
NP_077327.1	Heat-shock cognate 71 kDa protein	nucleus, cell membrane, cytoplasm	chaperone, stress response, aging, transcription
NP_059023.1	Guanine nucleotide-binding protein G(o) subunit alpha	cell membrane	transducer, AC-modulating G-protein-coupled receptor, signaling pathway, aging
P62260.1	14-3-3 protein epsilon	nucleus, cytoplasm	regulatory factor, signal transduction, brain development, MAPK cascade
NP_037150.2	Ras-related protein Rab-3A	cell membrane	exocytosis, axonogenesis, protein transport
NP_997477.1	Lactoylglutathione lyase	cytoplasm, nucleus, cell membrane	pyruvate metabolism, negative regulation of apoptosis
NP_058932.1	Phosphatidylethanolamine-binding protein 1	cytoplasm, cell membrane	protease inhibitor, aging, MAPK cascade, response to wounding, response to oxidative stress
NP_075211.2	Triosephosphate isomerase	cytoplasm	glycolysis, gluconeogenesis
NP_446090.1	Isocitrate dehydrogenase (NAD) subunit alpha, mitochondrial precursor	mitochondrion	Krebs cycle
NP_150238.1	Malate dehydrogenase, cytoplasmic isoform MDH1	cytoplasm	Krebs cycle
NP_058933.2	Ubiquitin carboxyl-terminal hydrolase isozyme L1	cytoplasm, endoplasmic reticulum, cell membrane	ubiquitin conjugation pathway, possible role in protecting cells under stress condition
NP_476484.1	Parkinson disease protein 7 homolog isoform 2	cell membrane, cytoplasm, nucleus, mitochondrion	chaperone, negative regulation of apoptosis, aging, response to oxidative stress, inflammatory response

**Table 3:** LC–MS/MS analysis of altered protein spots in PNS fractions isolated from mPFC of SNL versus sham-operated rats 8 weeks after the surgery. The complete list of exclusive unique peptides is presented in Table S2.

Spot	Accession Number	Protein	Exclusive Unique Peptides	SC ^a^ (%)	MW (kDa)	pI ^b^	Change ^c^ (Fold)	*p*-Value
1	NP_001004279.1	Peptidyl-prolyl *cis–trans* isomerase D	17	49	41	7.27	↑2.1	0.0267
2	NP_058704.1	Glyceraldehyde-3-phosphate dehydrogenase	16	70	36	8.87	↑2.1	0.0386
3	NP_075211.2	Triosephosphate isomerase	14	68	27	7.20	↑2.0	0.0370
4	XP_006252159.1	Dihydropyrimidinase-related protein 2 isoform X1	17	52	73	5.87	↑2.2	0.0216

^a^ Sequence coverage; ^b^ isoelectric point; ^c^ in SNL rats compared to sham rats.

**Table 4:** Subcellular localization and function of altered proteins in mPFC isolated from SNL versus sham-operated rats 8 weeks after the surgery.

Accession Number	Protein	Subcellular Localization	Molecular Functions and Biological Processes
NP_001004279.1	Peptidyl-prolyl *cis–trans* isomerase D	nucleus, cytoplasm	chaperone, negative regulation of apoptosis, protein transport, response to oxidative stress
NP_058704.1	Glyceraldehyde-3-phosphate dehydrogenase	cytoplasm, nucleus	glycolysis, response to oxidative stress, apoptosis, regulation of translation
NP_075211.2	Triosephosphate isomerase	cytoplasm	glycolysis, gluconeogenesis
XP_006252159.1	Dihydropyrimidinase-related protein 2 isoform X1	cytoplasm, cell membrane	developmental protein, remodeling of the cytoskeleton after injury, differentiation, neurogenesis

## Data Availability

The mass spectrometry proteomics data were deposited to the ProteomeXchange Consortium via the PRIDE partner repository under dataset identifiers.

## Supplementary Materials

The following are available online https://www.mdpi.com/article/10.3390/ijms23020955/s1.
