# Supplementary material for: Time-Dependent Changes in Protein Composition of Medial Prefrontal Cortex in Rats with Neuropathic Pain
Source: Int J Mol Sci. 2022 Jan 16;23(2):955. doi: 10.3390/ijms23020955 (PMC8781622; doi:10.3390/ijms23020955)
Supplement: Supplementary file 1 [file ijms-23-00955-s001.zip › Table S1.pdf]

**Table S1. LC-MS/MS analysis of altered protein spots in PNS fractions isolated from mPFC of SNL-treated versus sham-treated rats 2 weeks after the surgery; list of exclusive unique peptides used for protein identification.**

| Spot | Accession number | Protein name | Exclusive unique peptides | Modifications | SC [a] [%] | MW [b] (kDa) | pI [c] |
|---|---|---|---|---|---|---|---|
| 1 | NP_077327.1 | Heat shock cognate 71 kDa protein | (K)GPAVGIDLGTTYScVGVFQHGK(V) | Carbamidomethyl (+57) | 69 | 71 | 5.47 |
| | | | (K)NQVAmNPTNTVFDAK(R) | Oxidation (+16) | | | |
| | | | (R)RFDDAVVQSDMK(H) | | | | |
| | | | (R)FDDAVVQSDmK(H) | Oxidation (+16) | | | |
| | | | (K)HWPFMVVNDAGRPK(V) | | | | |
| | | | (K)VQVEYKGETK(S) | | | | |
| | | | (K)SFYPEEVSSMVLTK(M) | | | | |
| | | | (K)mKEIAEAYLGK(T) | Oxidation (+16) | | | |
| | | | (K)EIAEAYLGK(T) | | | | |
| | | | (K)TVTNAVVTVPAYFNDSQR(Q) | | | | |
| | | | (K)DAGTIAGLNVLR(I) | | | | |
| | | | (R)MVNHFIAEFK(R) | | | | |
| | | | (R)TLSSSTQASIEIDSLYEGIDFYTSITR(A) | | | | |
| | | | (K)SQIHDIVLVGGSTR(I) | | | | |
| | | | (K)SINPDEAVAYGAAVQAAILSGDK(S) | | | | |
| | | | (K)FELTGIPPAPR(G) | | | | |
| | | | (K)NSLESYAFNMK(A) | | | | |
| | | | (K)cNEIISWLDK(N) | Carbamidomethyl (+57) | | | |
| | | | (K)cNEIISWLDKNQTAEKEEFEHQQK(E) | Carbamidomethyl (+57) | | | |
| | | | (K)LYQSAGGMPGGMPGGFPGGGAPPSGGASSGPTIEEVD(-) | | | | |
| | | | (R)mVNHFIAEFK(R) | Oxidation (+16) | | | |
| | | | (K)SFYPEEVSSmVLTK(M) | Oxidation (+16) | | | |
| | | | (K)SQIHDIVLVGGSTR(I) | | | | |
| 2 | NP_059023.1 | Guanine nucleotide-binding protein G(O) subunit alpha | (K)NLKEDGISAAK(D) | | 58 | 40 | 5.48 |
| | | | (K)IIHEDGFSGEDVK(Q) | | | | |
| | | | (R)AMDTLGVEYGDK(E) | | | | |
| | | | (R)AMDTLGVEYGDKER(K) | | | | |
| | | | (R)AmDTLGVEYGDKER(K) | Oxidation (+16) | | | |
| | | | (R)MEDTEPFSAELLSAMMR(L) | | | | |
| | | | (R)MEDTEPFSAELLSAmMR(L) | Oxidation (+16) | | | |

| | | | | | | | |
|---|---|---|---|---|---|---|---|
| | | | (R)MEDTEPFSAELLSAMmR(L) | Oxidation (+16) | | | |
| | | | (R)LWGDSGIQEcFNR(S) | Carbamidomethyl (+57) | | | |
| | | | (R)SREYQLNDSAK(Y) | | | | |
| | | | (R)EYQLNDSAK(Y) | | | | |
| | | | (R)IGAADYQPTEQDILR(T) | | | | |
| | | | (R)MHESLMLFDSIcNNK(F) | Carbamidomethyl (+57) | | | |
| | | | (K)FFIDTSIILFLNK(K) | | | | |
| | | | (K)KSPLTIcFPEYPGSNTYEDAAAYIQTQFESK(N) | Carbamidomethyl (+57) | | | |
| | | | (K)SPLTIcFPEYPGSNTYEDAAAYIQTQFESK(N) | Carbamidomethyl (+57) | | | |
| 3 | P62260.1 | 14-3-3 protein epsilon | (-)mDDREDLVYQAK(L) | Acetyl (+42) | 84 | 29 | 4.86 |
| | | | (K)LAEQAERYDEMVESMK(K) | | | | |
| | | | (R)YDEMVESMK(K) | | | | |
| | | | (R)YDEMVESmK(K) | Oxidation (+16) | | | |
| | | | (K)KVAGMDVELTVEER(N) | | | | |
| | | | (K)VAGMDVELTVEER(N) | | | | |
| | | | (K)VAGmDVELTVEER(N) | Oxidation (+16) | | | |
| | | | (K)LIccDILDVLDK(H) | Carbamidomethyl (+57), Carbamidomethyl (+57) | | | |
| | | | (K)LIccDILDVLDKHLIPAANTGESK(V) | Carbamidomethyl (+57), Carbamidomethyl (+57) | | | |
| | | | (K)HLIPAANTGESK(V) | | | | |
| | | | (R)YLAEFATGNDR(K) | | | | |
| | | | (R)YLAEFATGNDRK(E) | | | | |
| | | | (K)EAAENSLVAYK(A) | | | | |
| | | | (K)AASDIAMTELPPTHPIR(L) | | | | |
| | | | (K)AAFDDAIAELDTLSEESYK(D) | | | | |
| | | | (R)DNLTLWTSDMQGDGEEQNK(E) | | | | |
| | | | (R)DNLTLWTSDMQGDGEEQNKEALQDVEDENQ(-) | | | | |
| | | | (K)EALQDVEDENQ(-) | | | | |
| 4 | NP_037150.2 | Ras-related protein Rab-3A | (K)ESSDQNFDYMFK(I) | | 49 | 25 | 4.73 |
| | | | (R)YADDSFTPAFVSTVGIDFK(V) | | | | |
| | | | (K)TYSWDNAQVLLVGNK(C) | | | | |
| | | | (R)QLADHLGFEFFEASAK(D) | | | | |
| | | | (K)MSESLDTADPAVTGAK(Q) | | | | |
| | | | (K)mSESLDTADPAVTGAK(Q) | Oxidation (+16) | | | |
| 5 | NP_997477.1 | lactoylglutathione lyase | (K)DFLLQQTmLR(I) | Oxidation (+16) | 56 | 20.8 | 5.13 |

| | | | | | | | |
|---|---|---|---|---|---|---|---|
| | | | (K)DFLLQQTMLR(I) | | | | |
| | | | (R)VLGLTLLQK(L) | | | | |
| | | | (K)FSLYFLAYEDK(N) | | | | |
| | | | (K)FSLYFLAYEDKNDIPK(D) | | | | |
| | | | (K)FSLYFLAYEDKNDIPKDK(T) | | | | |
| | | | (K)ATLELTHNWGTEDDETQSYHNGNSDPR(G) | | | | |
| | | | (R)GFGHIGIAVPDVYEAcK(R) | Carbamidomethyl (+57) | | | |
| | | | (R)GFGHIGIAVPDVYEAcKR(F) | Carbamidomethyl (+57) | | | |
| | | | (K)GLAFVQDPDGYWIEILNPNK(M) | | | | |
| 6 | NP_058932.1 | phosphatidylethanolamine-binding protein 1 | (R)VDYGGVTVDELGK(V) | | 62 | 20.8 | 5.47 |
| | | | (K)VLTPTQVmNRPSSISWDGLDPGK(L) | Oxidation (+16) | | | |
| | | | (K)LYTLVLTDPDAPSR(K) | | | | |
| | | | (K)LYTLVLTDPDAPSRK(D) | | | | |
| | | | (K)GNDISSGTVLSEYVGSGPPK(D) | | | | |
| | | | (K)GNDISSGTVLSEYVGSGPPKDTGLHR(Y) | | | | |
| | | | (R)YVWLVYEQEQPLNcDEPILSNK(S) | Carbamidomethyl (+57) | | | |
| | | | (K)YHLGAPVAGTcFQAEWDDSVPK(L) | Carbamidomethyl (+57) | | | |
| 7 | NP_075211.2 | triosephosphate isomerase | (K)cLGELIcTLNAAK(L) | Carbamidomethyl (+57), Carbamidomethyl (+57) | 71 | 26.9 | 7.37 |
| | | | (K)LPADTEVVcAPPTAYIDFAR(Q) | Carbamidomethyl (+57) | | | |
| | | | (K)IAVAAQNcYK(V) | Carbamidomethyl (+57) | | | |
| | | | (K)VTNGAFTGEISPGMIK(D) | | | | |
| | | | (K)VTNGAFTGEISPGmIK(D) | Oxidation (+16) | | | |
| | | | (K)DLGATWVVLGHSER(R) | | | | |
| | | | (R)RHIFGESDELIGQK(V) | | | | |
| | | | (R)HIFGESDELIGQK(V) | | | | |
| | | | (K)VNHALSEGLGVIAcIGEK(L) | Carbamidomethyl (+57) | | | |
| | | | (K)VVLAYEPVWAIGTGK(T) | | | | |
| | | | (K)TATPQQAQEVHEK(L) | | | | |
| | | | (K)cNVSEGVAQcTR(I) | Carbamidomethyl (+57), Carbamidomethyl (+57) | | | |
| | | | (R)IIYGGSVTGATcK(E) | Carbamidomethyl (+57) | | | |
| | | | (K)ELASQPDVDGFLVGGASLKPEFVDIINAK(-) | | | | |
| 8 | NP_075211.2 | triosephosphate isomerase | (K)cLGELIcTLNAAK(L) | Carbamidomethyl (+57), Carbamidomethyl (+57) | 64 | 26.9 | 7.59 |
| | | | (K)IAVAAQNcYK(V) | Carbamidomethyl (+57) | | | |

| | | | | | | | |
|---|---|---|---|---|---|---|---|
| | | | (K)VTNGAFTGEISPGMIK(D) | | | | |
| | | | (K)VTNGAFTGEISPGmIK(D) | Oxidation (+16) | | | |
| | | | (K)DLGATWVVLGHSER(R) | | | | |
| | | | (R)RHIFGESDELIGQK(V) | | | | |
| | | | (R)HIFGESDELIGQK(V) | | | | |
| | | | (K)VNHALSEGLGVIAcIGEK(L) | Carbamidomethyl (+57) | | | |
| | | | (K)VVLAYEPVWAIGTGK(T) | | | | |
| | | | (K)TATPQQAQEVHEK(L) | | | | |
| | | | (K)cNVSEGVAQcTR(I) | Carbamidomethyl (+57), Carbamidomethyl (+57) | | | |
| | | | (R)IIYGGSVTGATcK(E) | Carbamidomethyl (+57) | | | |
| | | | (K)ELASQPDVDGFLVGGASLKPEFVDIINAK(-) | | | | |
| 9 | NP_446090.1 | isocitrate dehydrogenase [NAD] subunit alpha, mitochondrial precursor | (R)NVTAIQGPGGK(W) | | 46 | 39.6 | 6.13 |
| | | | (K)TPIAAGHPSMNLLLR(K) | | | | |
| | | | (K)TPIAAGHPSmNLLLR(K) | Oxidation (+16) | | | |
| | | | (K)TFDLYANVRPcVSIEGYK(T) | Carbamidomethyl (+57) | | | |
| | | | (K)TPYTDVNIVTIR(E) | | | | |
| | | | (R)ENTEGEYSGIEHVIVDGVVQSIK(L) | | | | |
| | | | (K)RIAEFAFEYAR(N) | | | | |
| | | | (R)IAEFAFEYAR(N) | | | | |
| | | | (R)mSDGLFLQK(C) | Oxidation (+16) | | | |
| | | | (R)MSDGLFLQK(C) | | | | |
| | | | (K)DmANPTALLLSAVmMLR(H) | Oxidation (+16), Oxidation (+16) | | | |
| | | | (K)DMANPTALLLSAVmMLR(H) | Oxidation (+16) | | | |
| | | | (R)HmGLFDHAAK(I) | Oxidation (+16) | | | |
| | | | (K)IEAAcFATIK(D) | Carbamidomethyl (+57) | | | |
| | | | (K)cSDFTEEIcR(R) | Carbamidomethyl (+57), Carbamidomethyl (+57) | | | |
| 10 | NP_150238.1 | malate dehydrogenase, cytoplasmic isoform MDH1 | (K)DLDVAVLVGSMPR(R) | | 50 | 36.5 | 6.41 |
| | | | (K)DLDVAVLVGSmPR(R) | Oxidation (+16) | | | |
| | | | (K)VIVVGNPANTNcLTASK(S) | Carbamidomethyl (+57) | | | |
| | | | (K)LGVTADDVK(N) | | | | |
| | | | (K)NVIIWGNHSSTQYPDVNHAK(V) | | | | |
| | | | (K)EVGVYEALK(D) | | | | |

| | | | | | | | |
|---|---|---|---|---|---|---|---|
| | | | (K)EVGVYEALKDDSWLK(G) | | | | |
| | | | (K)GEFITTVQQR(G) | | | | |
| | | | (K)LSSAMSAAK(A) | | | | |
| | | | (R)DIWFGTPEGEFVSmGVISDGNSYGVPDDLLYSFPVVIK(N) | Oxidation (+16) | | | |
| 11 | NP_058933.2 | Ubiquitin carboxyl-terminal hydrolase isozyme L1 | (-)MQLKPmEINPEMLNK(V) | Oxidation (+16) | 87 | 24.8 | 5.24 |
| | | | (-)MQLKPmEINPEmLNK(V) | Oxidation (+16), Oxidation (+16) | | | |
| | | | (-)mQLKPmEINPEmLNK(V) | Oxidation (+16), Oxidation (+16), Oxidation (+16) | | | |
| | | | (-)mQLKPmEINPEMLNK(V) | Acetyl (+42), Oxidation (+16) | | | |
| | | | (R)FADVLGLEEETLGSVPSPAcALLLLFPLTAQHENFR(K) | Carbamidomethyl (+57) | | | |
| | | | (K)QIEELKGQEVSPK(V) | | | | |
| | | | (K)QTIGNScGTIGLIHAVANNQDK(L) | Carbamidomethyl (+57) | | | |
| | | | (K)QTIGNScGTIGLIHAVANNQDKLEFEDGSVLK(Q) | Carbamidomethyl (+57) | | | |
| | | | (K)LEFEDGSVLK(Q) | | | | |
| | | | (K)LEFEDGSVLKQFLSETEK(L) | | | | |
| | | | (K)NEAIQAAHDSVAQEGQcR(V) | Carbamidomethyl (+57) | | | |
| | | | (R)VDDKVNFHFILFNNVDGHLYELDGR(M) | | | | |
| | | | (K)VNFHFILFNNVDGHLYELDGR(M) | | | | |
| | | | (R)mPFPVNHGASSEDSLLQDAAK(V) | Oxidation (+16) | | | |
| | | | (R)EFTEREQGEVR(F) | | | | |
| 12 | NP_476484.1 | Parkinson disease protein 7 homolog isoform 2 | (K)GAEEMETVIPVDIMR(R) | | 72 | 20 | 6.73 |
| | | | (K)GAEEmETVIPVDIMR(R) | Oxidation (+16) | | | |
| | | | (K)GAEEMETVIPVDImR(R) | Oxidation (+16) | | | |
| | | | (K)VTVAGLAGK(D) | | | | |
| | | | (K)VTVAGLAGKDPVQcSR(D) | Carbamidomethyl (+57) | | | |
| | | | (R)DVVIcPDTSLEEAK(T) | Carbamidomethyl (+57) | | | |
| | | | (K)TQGPYDVVVLPGGNLGAQNLSESALVK(E) | | | | |
| | | | (R)KGLIAAIcAGPTALLAHEVGFGcK(V) | Carbamidomethyl (+57) | | | |
| | | | (R)GPGTSFEFALAIVEALSGK(D) | Carbamidomethyl (+57) | | | |

[a] the percentage of the sequence covered by identifications from the included searches
[b] the molecular weight of the protein
[c] the observed isoelectric point value
