# Supplementary material for: Time-Dependent Changes in Protein Composition of Medial Prefrontal Cortex in Rats with Neuropathic Pain
Source: Int J Mol Sci. 2022 Jan 16;23(2):955. doi: 10.3390/ijms23020955 (PMC8781622; doi:10.3390/ijms23020955)
Supplement: Supplementary file 1 [file ijms-23-00955-s001.zip › Table S2.pdf]

**Table S2. LC-MS/MS analysis of altered protein spots in PNS fractions isolated from the mPFC of SNL-treated versus sham-treated rats 8 weeks after the surgery; list of exclusive unique peptides used for identification.**

| Spot | Accession number | Protein name | Exclusive unique peptides | Modifications | SC [a] [%] | MW [b] (kDa) | pI [c] |
|---|---|---|---|---|---|---|---|
| 1 | NP_001004279.1 | peptidyl-prolyl cis-trans isomerase | (R)VFFDVDIGGER(V) | | 49 | 40.7 | 7.27 |
| | | | (R)IVLELFADIVPK(T) | | | | |
| | | | (K)HVVFGQVIK(G) | | | | |
| | | | (R)mLENVEVNGEKPAK(L) | Oxidation (+16) | | | |
| | | | (R)MLENVEVNGEKPAK(L) | | | | |
| | | | (K)LcVIAEcGELK(E) | Carbamidomethyl (+57), Carbamidomethyl (+57) | | | |
| | | | (K)LcVIAEcGELKEGDEWGIFPK(D) | Carbamidomethyl (+57), Carbamidomethyl (+57) | | | |
| | | | (K)DGSGDSHPDFPEDADIDLK(D) | | | | |
| | | | (K)DGSGDSHPDFPEDADIDLKDVDK(I) | | | | |
| | | | (K)ILLISEDLK(N) | | | | |
| | | | (K)NIGNTFFK(S) | | | | |
| | | | (R)LQPIALScVLNIGAcK(L) | Carbamidomethyl (+57), Carbamidomethyl (+57) | | | |
| | | | (K)MSNWQGAIDScLEALEMDPSNTK(A) | Carbamidomethyl (+57) | | | |
| | | | (K)mSNWQGAIDScLEALEMDPSNTK(A) | Oxidation (+16), Carbamidomethyl (+57) | | | |
| | | | (K)AQGWQGLK(E) | | | | |
| | | | (K)EYDQALADLK(K) | | | | |
| | | | (K)EYDQALADLKK(A) | | | | |
| 2 | NP_058704.1 | glyceraldehyde-3-phosphate dehydrogenase | (K)VDIVAINDPFIDLNYmVYMFQYDSTHGK(F) | Oxidation (+16) | 70 | 35.8 | 8.87 |
| | | | (K)VDIVAINDPFIDLNYmVYmFQYDSTHGK(F) | Oxidation (+16), Oxidation (+16) | | | |
| | | | (K)LVINGKPITIFQER(D) | | | | |
| | | | (K)LVINGKPITIFQERDPANIK(W) | | | | |
| | | | (R)DPANIKWGDAGAEYVVESTGVFTTmEK(A) | Oxidation (+16) | | | |
| | | | (K)WGDAGAEYVVESTGVFTTMEK(A) | | | | |
| | | | (K)WGDAGAEYVVESTGVFTTmEK(A) | Oxidation (+16) | | | |
| | | | (K)RVIISAPSADAPmFVmGVNHEK(Y) | Oxidation (+16), Oxidation (+16) | | | |
| | | | (K)IVSNAScTTNcLAPLAK(V) | Carbamidomethyl (+57), Carbamidomethyl (+57) | | | |
| | | | (K)VIHDNFGIVEGLmTTVHAITATQK(T) | Oxidation (+16) | | | |
| | | | (R)GAAQNIIPASTGAAK(A) | | | | |
| | | | (R)VPTPNVSVVDLTcR(L) | Carbamidomethyl (+57) | | | |

| | | | | | | | |
|---|---|---|---|---|---|---|---|
| | | | (K)GILGYTEDQVVScDFNSNSHSSTFDAGAGIALNDNFVK(L) | Carbamidomethyl (+57) | | | |
| | | | (K)LISWYDNEYGYSNR(V) | | | | |
| | | | (R)VVDLmAYMASK(-) | Oxidation (+16) | | | |
| | | | (R)VVDLmAYmASKE(-) | Oxidation (+16), Oxidation (+16) | | | |
| 3 | NP_075211.2 | triosephosphate isomerase | (K)cLGELIcTLNAAK(L) | Carbamidomethyl (+57), Carbamidomethyl (+57) | 68 | 26.9 | 7.20 |
| | | | (K)LPADTEVVcAPPTAYIDFAR(Q) | Carbamidomethyl (+57) | | | |
| | | | (K)IAVAAQNcYK(V) | Carbamidomethyl (+57) | | | |
| | | | (K)VTNGAFTGEISPGMIK(D) | | | | |
| | | | (K)VTNGAFTGEISPGmIK(D) | Oxidation (+16) | | | |
| | | | (K)DLGATWVVLGHSER(R) | | | | |
| | | | (R)RHIFGESDELIGQK(V) | | | | |
| | | | (R)HIFGESDELIGQK(V) | | | | |
| | | | (K)VNHALSEGLGVIAcIGEK(L) | Carbamidomethyl (+57) | | | |
| | | | (K)VVLAYEPVWAIGTGK(T) | | | | |
| | | | (K)TATPQQAQEVHEK(L) | | | | |
| | | | (K)cNVSEGVAQcTR(I) | Carbamidomethyl (+57), Carbamidomethyl (+57) | | | |
| | | | (R)IIYGGSVTGATcK(E) | Carbamidomethyl (+57) | | | |
| | | | (K)ELASQPDVDGFLVGGASLKPEFVDIINAK(-) | | | | |
| 4 | XP_006252159.1 | dihydropyrimidinase-related protein 2 isoform X1 | (R)mVIPGGIDVHTR(F) | Oxidation (+16) | 52 | 73.1 | 5.87 |
| | | | (R)FQmPDQGmTSADDFFQGTK(A) | Oxidation (+16), Oxidation (+16) | | | |
| | | | (K)GIQEEMEALVK(D) | | | | |
| | | | (K)DRFQLTDSQIYEVLSVIR(D) | | | | |
| | | | (R)FQLTDSQIYEVLSVIR(D) | | | | |
| | | | (R)DIGAIAQVHAENGDIIAEEQQR(I) | | | | |
| | | | (R)ILDLGITGPEGHVLSRPEEVEAEAVNR(S) | | | | |
| | | | (R)SITIANQTNcPLYVTK(V) | Carbamidomethyl (+57) | | | |
| | | | (K)SAAEVIAQAR(K) | | | | |
| | | | (K)GTVVYGEPITASLGTDGSHYWSK(N) | | | | |
| | | | (K)AAAFVTSPPLSPDPTTPDFLNSLLScGDLQVTGSAHcTFNTAQK(A) | Carbamidomethyl (+57), Carbamidomethyl (+57) | | | |
| | | | (K)DNFTLIPEGTNGTEER(M) | | | | |
| | | | (R)ISVGSDADLVIWDPDSVK(T) | | | | |
| | | | (K)THNSALEYNIFEGmEcR(G) | Oxidation (+16), Carbamidomethyl (+57) | | | |
| | | | (R)GSPLVVISQGK(I) | | | | |

| | | | | | | | |
|---|---|---|---|---|---|---|---|
| | | | (K)IVLEDGTLHVTEGSGR(Y) | | | | |
| | | | (R)KPFPDFVYK(R) | | | | |

[a] the percentage of the sequence covered by identifications from the included searches
[b] the molecular weight of the protein
[c] the observed isoelectric point value
